# Chemical Burn From Cinnamon Oil

**Published:** 2017-06-01

**Authors:** Margaret Connolly, Andrea Axtell, Sean Hickey, Ann Whalen, Lucy McNamara, Dorothy Albright, Jonathan Friedstat, Jeremy Goverman

**Affiliations:** Sumner Redstone Burn Center, Department of Surgery, Massachusetts General Hospital, Boston

**Keywords:** cinnamon oil, chemical burn, cinnamaldehyde, cinnamon, burn

## DESCRIPTION

A 68-year-old woman presented with an 8% total body surface area superficial, partial-thickness chemical burn to her bilateral posterior thighs and buttocks after spilling cinnamon oil onto her seat cushion and sitting on it for 2 hours. She self-treated with Neosporin (neomycin/polymyxin B/bacitracin), Vaseline, and Silvadene (silver sulfadiazine) for 8 days before presenting to our burn center with increasing pain.

## QUESTIONS

1. **What are the components of cinnamon oil?**
2. **How are people commonly exposed to cinnamon oil?**
3. **What are the reported adverse reactions to cinnamon oil exposure?**
4. **How are adverse reactions treated?**

## DISCUSSION

Cinnamon oil is an essential oil that is produced from steam distillation of the bark of *Cinnamomum* trees. It has been used for a variety of medical purposes including glucose and cholesterol management, as well as in wound care as an antimicrobial and anti-inflammatory agent.^1^ Commercial cinnamon bark oil is composed of approximately 80% cinnamaldehyde, 10% *trans*-cinnamic acid, and 10% eugenol.^1^^,^^2^ Research has demonstrated the ability of cinnamaldehyde to induce expression of antioxidant genes in human keratinocytes,^3^ as well as to have bactericidal activity against multidrug-resistant pathogens such as *Pseudomonas aeruginosa*.^4^

Although studies provide some evidence for the potential medical use of cinnamon oil, most people today are exposed to cinnamon oil for nonmedical reasons. It is frequently used in a diluted form for its aroma and as a flavoring agent in gum, candies, baked goods, beverages, toothpaste, and mouthwash.^1^^,^^5^

Given the prevalence of such products in daily life, it is not surprising that a variety of reactions to cinnamon oil have been described in the literature. Allergic contact stomatitis has been described as red and white partially erosive lesions in a patient who chewed cinnamon-flavored gum a few times weekly. These lesions disappeared upon discontinuation of the gum.^5^ Allergic contact dermatitis has been described as vesicular eczematous lesions in a variety of patients: employees and customers exposed to various spa products, creams, and ointments containing cinnamon oil,^6^ a baker with lesions on her face from a bakery that made cinnamon buns,^7^ and a woman with lesions on her buttocks from a cinnamon oil-containing vaginal suppository.^2^ In all such cases, the patients underwent allergy patch-testing that confirmed reactivity to cinnamaldehyde and/or other components of cinnamon oil. These patients typically have no reaction to cinnamon as a spice. In our literature review, there is only one other report of a contact burn from cinnamon oil and this occurred in a boy who sustained a second-degree burn after a vial of cinnamon oil broke in his pocket and the area was unwashed for 2 days.^8^ Our patient also had prolonged contact with undiluted cinnamon oil and suffered a similar chemical burn.

Treatment of exposure to cinnamon oil should include the removal of contaminated clothing and prompt irrigation with copious amounts of water. Patients with small or superficial appearing burns may be treated as outpatients, while larger, or more severe, burns may require hospitalization and treatment as for a typical thermal burn (moist wound dressing). Topical steroids may play a role for early, minor reactions.

Cinnamon bark oil is commonly used for flavoring, for fragrance, and for a variety of medicinal purposes. Even in its diluted form, the components of cinnamon bark oil have been demonstrated to cause hypersensitivity in some patients. This can present as a stomatitis or dermatitis with a positive patch test to cinnamon oil. Fortunately, cessation of exposure to the inflicting agent usually results in the resolution of these adverse reactions. The case presented here represents the second report of prolonged exposure to undiluted cinnamon oil resulting in a partial-thickness chemical burn.

## Figures and Tables

**Figure 1:**
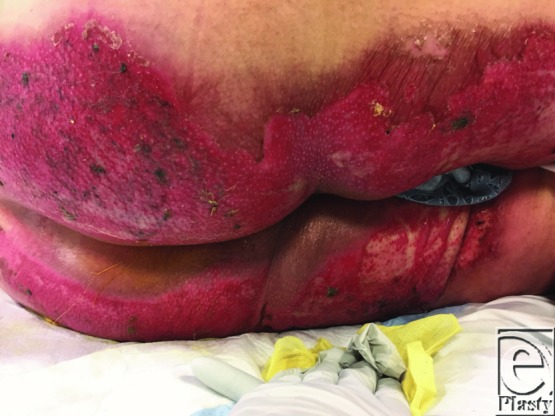
Superficial partial thickness burn from cinnamon oil, 8 days post injury.

**Figure 2:**
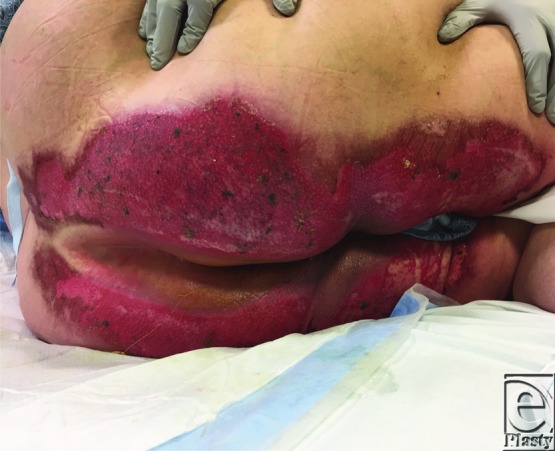
Superficial partial thickness burn from cinnamon oil, 8 days post injury.
